# GLP-1 receptor agonists alleviate inflammatory responses and endothelial dysfunction in atherosclerosis by activating the Sema3A/NRP1 pathway

**DOI:** 10.3389/fcvm.2026.1836833

**Published:** 2026-05-12

**Authors:** Lin Hu, Sanjun Li, Xiaoqin Deng, Zhipeng Zhou, Liu Yang, Bin Li

**Affiliations:** 1 Department of Cardiology, Jiangxi Provincial People’s Hospital (The First Affiliated Hospital of Nanchang Medical College), Nanchang, China; 2 General Practice, Nanchang University Hospital, Nanchang, China

**Keywords:** atherosclerosis, GLP-1 receptor agonists, NRP1, Sema3A, semaglutide, vascular endothelium

## Abstract

**Objective:**

This study aimed to investigate the mechanism by which GLP-1 RAs activate the Sema3A/NRP1 signaling pathway to alleviate inflammation and endothelial dysfunction in atherosclerosis (AS).

**Methods:**

Ten 8-week-old male C57BL/6J mice served as controls (CON) and received intraperitoneal injections of saline every 2 days. Forty *ApoE^−/−^* mice were fed a 60 kcal% fat high-fat diet for 12 weeks. From weeks 13 to 24, the *ApoE^−/−^* mice were randomly divided into 4 groups (*n* = 10): AS model group (AS, saline injections), low/high-dose GLP-1 RAs group (L/H-GLP-1 RAs, 30/60 μg/kg semaglutide injections) and atorvastatin group (ATO, 1.3 mg/kg atorvastatin injections as a positive control). For rescue experiments, endothelial-specific NRP1 knockout (*NRP1^EC-KO^*) mice were generated. Thirty *NRP1^WT^* mice were divided into 3 groups (*n* = 10): *NRP1^WT^* control group (*NRP1^WT^* CON), the *NRP1^WT^* AS model group (*NRP1^WT^* AS) and the *NRP1^WT^* AS model + high dose GLP-1 RAs group (*NRP1^WT^* + H-GLP-1 RAs). While twenty *NRP1^EC-KO^* mice were divided into *NRP1^EC-KO^* AS and *NRP1^EC-KO^* + H-GLP-1 RAs groups (*n* = 10). Mouse body weight was monitored weekly during interventions, and mice were euthanized with 120 mg/kg pentobarbital sodium at the end of experiments. HUVECs were divided into five groups (*n* = 3): CON, ox-LDL, L-GLP-1 RAs, H-GLP-1 RAs, ATO. Except for controls, cells were treated with 100 μg/mL ox-LDL for 24 h to establish the model. CCK-8 assays determined low/high semaglutide doses (1/2 μM), while atorvastatin was applied at 10 μM. Rescue experiments included CON, ox-LDL, H-GLP-1 RAs, and NRP1 inhibition + H-GLP-1 RAs groups, with the latter pre-treated with 0.5 μM NRP1 inhibitor EG01377 for 2 h before interventions.

**Results:**

Compared to controls, AS group mice exhibited significant weight gain from week 15 (*P* < 0.001), while H-GLP-1 RAs and ATO groups showed reduced weight from week 21 (*P* < 0.05). The AS group had elevated serum TC, LDL-C, IL-6, HDL-C, TNF-α, ET-1, TG levels, and percentage of plaque collagen-positive area (*P* < 0.001), alongside decreased NO levels (*P* < 0.001), all of which were improved by GLP-1 RAs (*P* < 0.05). H&E staining revealed reduced inflammatory cell infiltration in aortic roots of semaglutide- and atorvastatin-treated mice. Aortic tissues from AS group mice showed decreased Sema3A/NRP1 expression and binding (*P* < 0.05), increased p-ERK1/2/ERK1/2 and p-NF-κB p65/NF-κB p65 ratios (*P* < 0.05), which were reversed by semaglutide, with higher doses showing greater effects. Immunofluorescence confirmed that Sema3A and NRP1 mainly localized in vascular endothelial cells. In ox-LDL-induced HUVECs, GLP-1 RAs improved cell viability, migration capacity, and tubule numbers (*P* < 0.05), reduced IL-6 and TNF-α levels (*P* < 0.05), upregulated eNOS (*P* < 0.05), and downregulated VCAM-1 and ICAM-1 (*P* < 0.05). Western blot and co-immunoprecipitation results aligned with *in vivo* trends. *In vitro*, EG01377 reversed GLP-1 RAs' protective effects (*P* < 0.05). *In vivo*, GLP-1 RAs' therapeutic efficacy was significantly weakened in *NRP1^EC-KO^* mice (*P* < 0.05).

**Conclusion:**

GLP-1 RAs alleviate inflammation and endothelial dysfunction to reduce the atherosclerosis progression by activating the Sema3A/NRP1 pathway and inhibiting downstream ERK1/2-NF-κB signaling.

## 1 Introduction

Atherosclerosis (AS) represents the primary pathological basis of cardiovascular diseases. It can induce life-threatening complications such as myocardial infarction, cerebral infarction and renal atrophy, and has become a global public health burden (1). Excessive activation of inflammatory response and vascular endothelial dysfunction are the core pathological processes in the development of AS (2). Endothelial dysfunction compromises the integrity of vascular barrier, promoting lipid deposition and inflammatory cell infiltration, while the release of inflammatory factors further amplifies pathological damage and accelerates disease progression (3). The semaphorin (SEMA) family has now been widely recognized as a pivotal regulator of vascular homeostasis in both physiological and pathological contexts (4). Among them, Sema3A as a secreted glycoprotein, binds to the receptor complex composed of NRP1, a transmembrane glycoprotein receptor, and plexin A family members to regulate cell migration, proliferation and fibrosis (5). Evidence indicates that Sema3A expression is downregulated in hemodynamically sensitive regions of atherosclerotic lesions, suggesting that it may act as a potential protective factor (6). Meanwhile, NRP1 has been shown to regulate endothelial vascular permeability signaling (7), promote angiogenesis, and play a critical protective role in AS (8). Another study highlights that Sema3A and NRP1 exerts anti-atherosclerotic effects by maintaining endothelial junction integrity and suppressing pro-inflammatory gene transcription (9). These results collectively demonstrate that the Sema3A/NRP1 pathway may contributes to the regulation of angiogenesis, endothelial cell stability, and inflammatory responses, and is associated with the formation and progression of atherosclerotic plaques. Recent studies have shown that GLP-1 RAs exert cardioprotective effects independent of glucose-lowering effects (10). Research indicates that GLP-1 RAs significantly reduce the risk of adverse cardiovascular events in non-diabetic obese adults (11). The vasodilatory property of GLP-1 RAs improve endothelial function, while their anti-proliferative and anti-inflammatory effects may synergistically inhibit AS progression (12). However, whether the Sema3A/NRP1 signaling pathway mediates the cardiovascular protective effects of GLP-1 RAs remains unclear. Based on this, this study aims to investigate the molecular mechanisms by which GLP-1 RAs activate the Sema3A/NRP1 signaling pathway to alleviate inflammatory response and endothelial dysfunction, ultimately mitigating AS progression. Our findings may provide novel molecular targets for clinical treatment of AS and offer theoretical support for the cardiovascular protective effects of GLP-1 RAs.

## 2 Materials and methods

### 2.1 Chemicals and reagents

Semaglutide (HY-114118, CAS: 910463-68-2), atorvastatin (ATO, HY-B0589, CAS: 134523-00-5), NRP1 inhibitor EG01377 (HY-112151, CAS: 2227996-00-9) were purchased from MedChemExpress (Shanghai, China). Ox-LDL with purity ≥ 98% (IO1300) was obtained from Solarbio (Beijing, China). TC (A111-1-1), LDL-C (A113-1-1), IL-6 (H007-1-2), NO (A013-2-1), TG (A110-1-1), TNF-α (H052-1-2), ET-1 (H093-1-2) and HDL-C (A112-1-1) assay kit were procured from Nanjing Jiancheng (Jiangsu, China). H&E staining kit (C0105S), RIPA lysis buffer (P0013B), BCA assay kit (P0010S), antibody crosslink immunoprecipitation kit with protein A + G magnetic beads (P2180S), oil red O staining kit (C0157S), hypersensitive chemiluminescent liquid (ECL) kit (P0018S), DAPI staining solution (C1006) and matrix-gel™ basement membrane matrix (C0372) were procured from Beyotime (Shanghai, China). Anti-Sema3A (ab199475), anti-α-SMA (ab124964), anti-NF-κB p65 (ab32536), anti-ICAM-1 (ab171123), anti-GAPDH (ab181602), anti-p-NF-κB p65 (ab76302), anti-IgG (ab133470), anti-CD31 (ab222783), anti-eNOS (ab199956), anti-VCAM-1 (ab134047) and anti-NRP1 (ab81321) antibody were supplied by Abcam (Shanghai, China). Anti-p-ERK1/2 antibody (4370T) was provided by Cell Signaling Technology (Shanghai, China). PBS (10010023), DMEM (11965092) and 0.25% trypsin digestion solution (25200056) were supplied by ThermoFisher (Shanghai, China). CCK-8 kit (CK04) was sourced from Dojindo (Shanghai, China).

### 2.2 Animals and treatments

Eight-week-old male C57BL/6J mice and apolipoprotein E deficient (*ApoE^−/−^*) mice (20–22 g) were obtained from Charles River (Beijing, China). Experimental animal production license No. SCXK (Jing) 2021-0006. Mice were transferred to an SPF-grade animal facility for a 7-day acclimatization period. During this period, all mice were maintained under a 12-h light/dark cycle with free access to water and standard maintenance diet, and no experimental interventions were performed. After the 7-day acclimatization period, the experiment officially commenced. Ten C57BL/6J mice in the control group (CON) continuing to be fed the standard maintenance diet, while forty *ApoE^−/−^* mice were divided into 4 groups (*n* = 10): the AS model group (AS), the low/high-dose GLP-1 RAs groups (L/H-GLP-1 RAs) and the atorvastatin group (ATO). All *ApoE^−/−^* mice was fed a high-fat diet (60 kcal % fat) for the initial 12 weeks of the experiment (13). No drug or vehicle injections were administered to any mice during this period. Only routine feeding, weekly body weight monitoring, and health assessments were conducted. The CON group and the AS group received intraperitoneal injection of normal saline every day during the 13–24 weeks of the experiment. Similarly, the L/H-GLP-1 RAs groups received intraperitoneal injections of 30/60 μg/kg semaglutide every day, respectively (14), while the ATO group received intraperitoneal injection of 1.3 mg/kg/day atorvastatin every 2 days as the positive control (15). The U.S. Food and Drug Administration specifies that the standard dose of semaglutide for adult patients (70 kg) is 1–1.5 mg per week (subcutaneous injection, approximately 0.0020–0.0031 mg/kg/day), with a maximum approved dose of 2.4 mg per week (approximately 0.0049 mg/kg/day). Based on standard animal-to-human dose conversion (mouse conversion factor = 12.3), the corresponding mouse equivalent doses are approximately 25.1–37.7 and 60.2 μg/kg/day, respectively. The dose used in this study is similar to the clinical human equivalent dose. During the intervention period, body weight changes were monitored weekly. On the final day of the experiment, mice were humanely euthanized by intraperitoneal administration of 120 mg/kg sodium pentobarbital, followed by the collection of aortic root tissues and serum samples. All animal experiments were performed in compliance with the animal ethics guidelines of Jiangxi Provincial People's Hospital (The First Affiliated Hospital of Nanchang Medical College), including the ARRIVE 2.0 guidelines and the Chinese National Standard GB/T 35892-2018, Approval number: JX2024092.

### 2.3 Construction of endothelium-specific NRP1 knockout mice

*Tie2-Cre^+^* mice and *NRP1^flox/flox^* mice were obtained from Cyagen Biosciences (Suzhou, China). Experimental animal production license No. SYXK (Yue) 2025-0242. The primers used for mouse genotyping are provided as follows. *NRP1^flox^* forward primer: AAGGAGTGGCACAGCATCTT, *NRP1^flox^* reverse primer: TCACACCCAAACTTCCTTCC; *Tie2-Cre^+^* forward primer: TAGGAACCAATGAAATGCGAGGT, *Tie2-Cre^+^* reverse primer: AACCAGCGTTTTCGTTCTGC. Endothelial-specific NRP1 knockout (*NRP1^EC-KO^*) mice were generated using the Cre/loxP gene editing system through crossbreeding of *ApoE^−/−^* mice with *NRP1^flox/flox^; Tie2-Cre^+^* mice. Successful endothelium-specific NRP1 knockout was confirmed via qPCR and the mice were subsequently used in experiments. Thirty *NRP1^WT^* mice were divided into *NRP1^WT^* control group (*NRP1^WT^* CON), *NRP1^WT^* AS model group (*NRP1^WT^* AS), and *NRP1^WT^* AS model + high-dose GLP-1 RAs group (*NRP1^WT^* + H-GLP-1 RAs) (*n* = 10). Twenty *NRP1^EC-KO^* mice were assigned to *NRP1^EC-KO^* AS model group (*NRP1^EC-KO^* AS) and *NRP1^EC-KO^* AS model + high-dose GLP-1 RAs group (*NRP1^EC-KO^* + H-GLP-1 RAs) (*n* = 10). Other intervention procedures followed those described in Section 2.2.

### 2.4 Cell culture and treatments

HUVECs were cultured in DMEM and maintained in a incubator at 37 °C with 5% CO_2_. Cells in the control group (CON) received standard culture conditions without additional treatment. Cells in the ox-LDL treatment group (ox-LDL) were exposed to 100 μg/mL ox-LDL for 24 h to induce cellular injury (16). Following successful model establishment, cells were treated with 0.1, 0.5, 1, 2, 5, or 10 μM semaglutide. Cell viability was assessed to determine optimal experimental concentrations. Based on the results, 1 μM semaglutide was selected for the low-dose GLP-1 RAs group (L-GLP-1 RAs) and 2 μM semaglutide for the high-dose GLP-1 RAs (H-GLP-1 RAs) group. The atorvastatin group (ATO) received 10 μM atorvastatin treatment as a positive control after model induction (17). Rescue experiments included the CON group, ox-LDL group, H-GLP-1 RAs group and the NRP1 inhibitor and high-dose GLP-1 RAs combined treatment group (NRP1 inhibition + H-GLP-1 RAs). For the latter group, cells were pretreated with 0.5 μM NRP1 inhibitor EG01377 for 2 h before exposure to ox-LDL and high-dose semaglutide (18). All cell experiments were independently repeated 3 times.

### 2.5 Biochemical indices inflammation factors and endothelial factor detection

NO, TC, LDL-C, IL-6, TG, TNF-α, ET-1 and HDL-C levels in mouse blood or serum and IL-6, TNF-α levels in HUVECs culture supernatant were measured. Absorbance values were read using Varioskan LUX microplate reader (ThermoFisher, Waltham, USA).

### 2.6 Oil red O staining

Mouse aortic root sections were stained with Oil Red O and hematoxylin, then mounted. Tissue morphology was observed and images captured under DM3000 optical microscope (Leica, Wetzlar, Germany).

### 2.7 H&E staining

Paraffin sections were stained with hematoxylin and eosin, then mounted with neutral balsam. Morphology and inflammatory cell infiltration in aortic root plaques were analyzed under an optical microscope.

### 2.8 WB analysis

Mouse aortic tissues or HUVECs were collected and lysed to prepare protein samples. Following standard blocking procedures, samples were incubated overnight with primary antibodies (1:1000). The next day, secondary antibodies (1:5000) were applied for 1 h, followed by ECL chemiluminescent detection. ImageJ software was used to quantify protein band intensities normalized to GAPDH.

### 2.9 Co-immunoprecipitation (CO-IP) assay

Protein extracts from mouse aortic tissues or HUVECs were incubated with protein A/G agarose beads and Sema3A antibody, then incubate overnight. The next day, samples were centrifuged at 3,000 rpm at 4 °C for 5 min to collect precipitates, which were then denatured. WB analysis was performed using NRP1 antibody (1:1000), with subsequent steps following those described in Section 2.8.

### 2.10 Immunofluorescence (IF) assay

Mouse aortic tissues were fixed and sectioned into 8 μm-thick frozen slices. After blocking, sections were incubated with Sema3A, NRP1, CD31 or α-SMA antibody at 4 °C overnight. The following day, corresponding fluorescent secondary antibodies (1:500) were applied for 1 h. Tissues were mounted after nuclear staining with DAPI, then mounted and observed under LSM 880 microscope (Zeiss, Auberkheim, Germany).

### 2.11 CCK-8 assay

HUVECs were seeded in 96-well plates. CCK-8 reagent was added to each well, followed by incubation for 1 h. The absorbance at 450 nm was measured using a microplate reader.

### 2.12 Wound healing assay

HUVECs were seeded in 6-well plates. Uniform scratches were created perpendicular to the bottom of the wells using a 200 μL tip, after which the designated treatments were applied. Images of the same field were captured under an optical microscope.

### 2.13 Tube formation assay

HUVECs were seeded in 96-well plates pre-coated with Matrigel matrix gel and subjected to experimental treatments. Cells were cultured for 6 h. Tube formation was observed under an optical microscope.

### 2.14 Statistical analysis

All experimental data were analyzed using SPSS 22.0. Quantitative data were expressed as mean ± standard deviation (SD). Pairwise comparisons between groups were conducted using the least significant difference *t*-test, and comparisons among multiple groups were performed with one-way analysis of variance. Statistical significance was defined as *P* < 0.05.

## 3 Results

### 3.1 GLP-1 RAs ameliorate atherosclerotic phenotypes and inflammatory responses in mice

**Figure 1:**
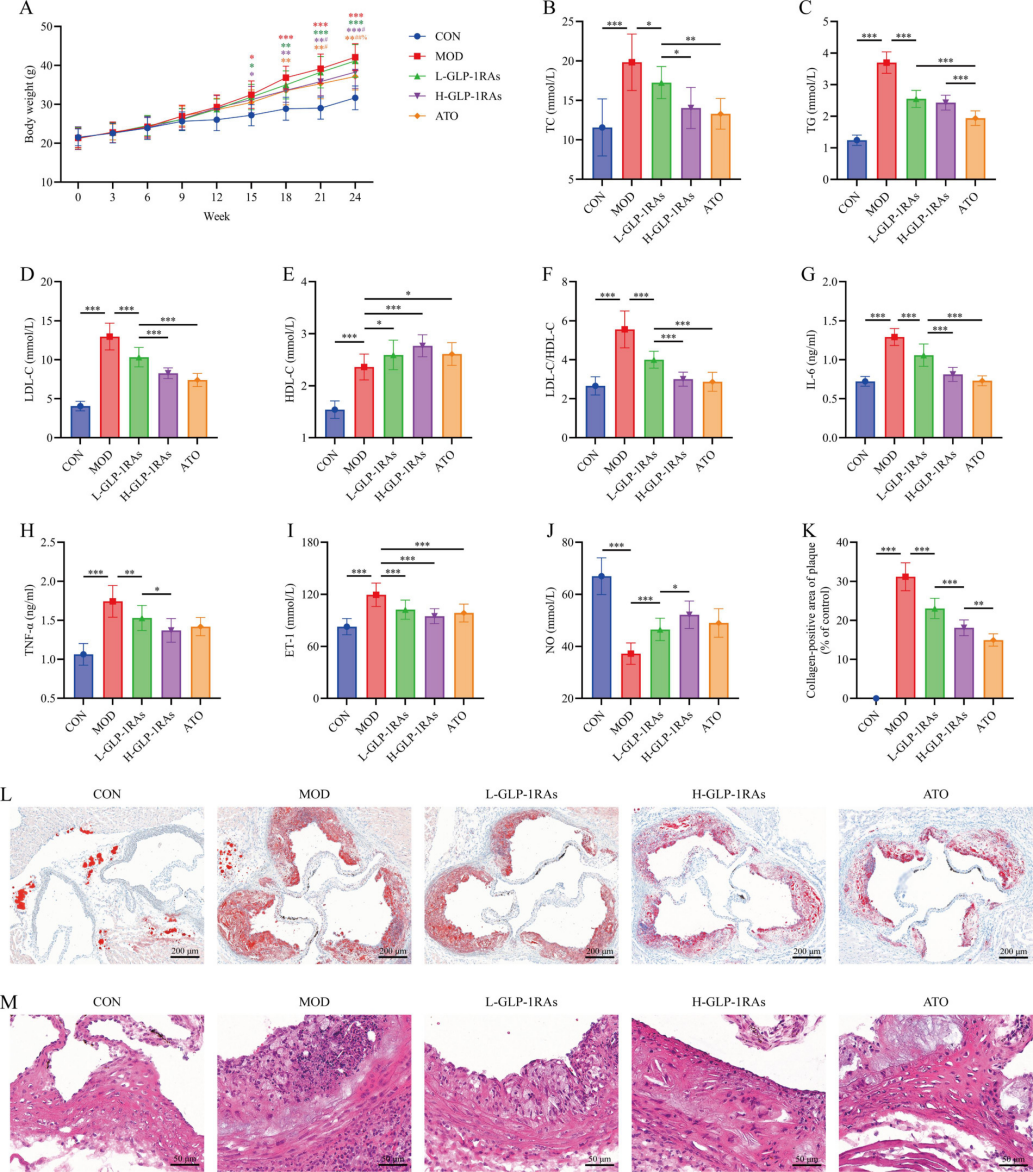
Effects of glucagon-like peptide-1 receptor agonists (GLP-1 RAs) on atherosclerotic (aS) phenotypes and inflammatory responses in mice. **(A)** Mouse body weight. *P < 0.05, **P < 0.01, ***P < 0.001 vs. CON group; ^#^*P* < 0.05, ^##^*P* < 0.01 vs. MOD group; ^%^*P* < 0.05 vs. L-GLP-1 RAs group. **(B)** Mouse triglycerides (TG) level. **(C)** Mouse total cholesterol (TC) level. **(D)** Mouse low-density lipoprotein cholesterol (LDL-C) level. **(E)** Mouse high-density lipoprotein cholesterol (HDL-C) level. **(F)** Mouse LDL-C/HDL-C ratio. **(G)** Mouse interleukin-6 (IL-6) level. **(H)** Mouse tumor necrosis factor-alpha (TNF-α) level. **(I)** Mouse endothelin-1 (ET-1) level. **(J)** Mouse nitric oxide (NO) level. **(K)** Percentage of collagen-positive area in mouse plaque. Collagen-positive area (%) = (collagen-positive area/plaque area) × 100%. **(L)** Oil red O staining of mouse aortic sinus. **(M)** Hematoxylin and eosin staining of mouse aortic sinus. The area outlined by the yellow box shows inflammatory cell infiltration. All data are presented as mean ± standard deviation. *n* = 10. CON, control group; AS, AS model group; L-GLP-1 RAs, low-dose GLP-1 RAs (semaglutide) group; H-GLP-1 RAs, high-dose GLP-1 RAs (semaglutide) group; ATO, atorvastatin group. *P < 0.05, **P < 0.01, ***P < 0.001.

Figure 1A showed that the body weight of MOD mice exhibited significantly higher body weights compared to CON mice from the 15th week (*P* < 0.001). Starting from week 21, both high dose semaglutide and ATO-treated mice showed significantly reduced body weights compared to MOD mice (*P* < 0.05). Figures 1B–L reveal that compared to the CON mice, MOD mice exhibited elevated levels of TC, ET-1, IL-6, TG, TNF-α, LDL-C/HDL-C and percentage of collagen-positive area in plaque (*P* < 0.001), alongside decreased NO levels (*P* < 0.001). Following semaglutide treatment at different doses, the above indicators were remarkably improved (*P* < 0.05). Histopathological analysis of aortic root sections using H&E staining (Figure 1M) revealed inflammatory cell infiltration in the atherosclerotic model mice, characterized by small cell size, deeply blue-stained nuclei, and scant cytoplasm (outlined in yellow), while semaglutide and ATO interventions reduced tissue inflammation.

### 3.2 GLP-1 RAs upregulate Sema3A/NRP1 expression and binding levels in aortic tissues of atherosclerotic mice and inhibit downstream ERK1/2 signaling activation

**Figure 2:**
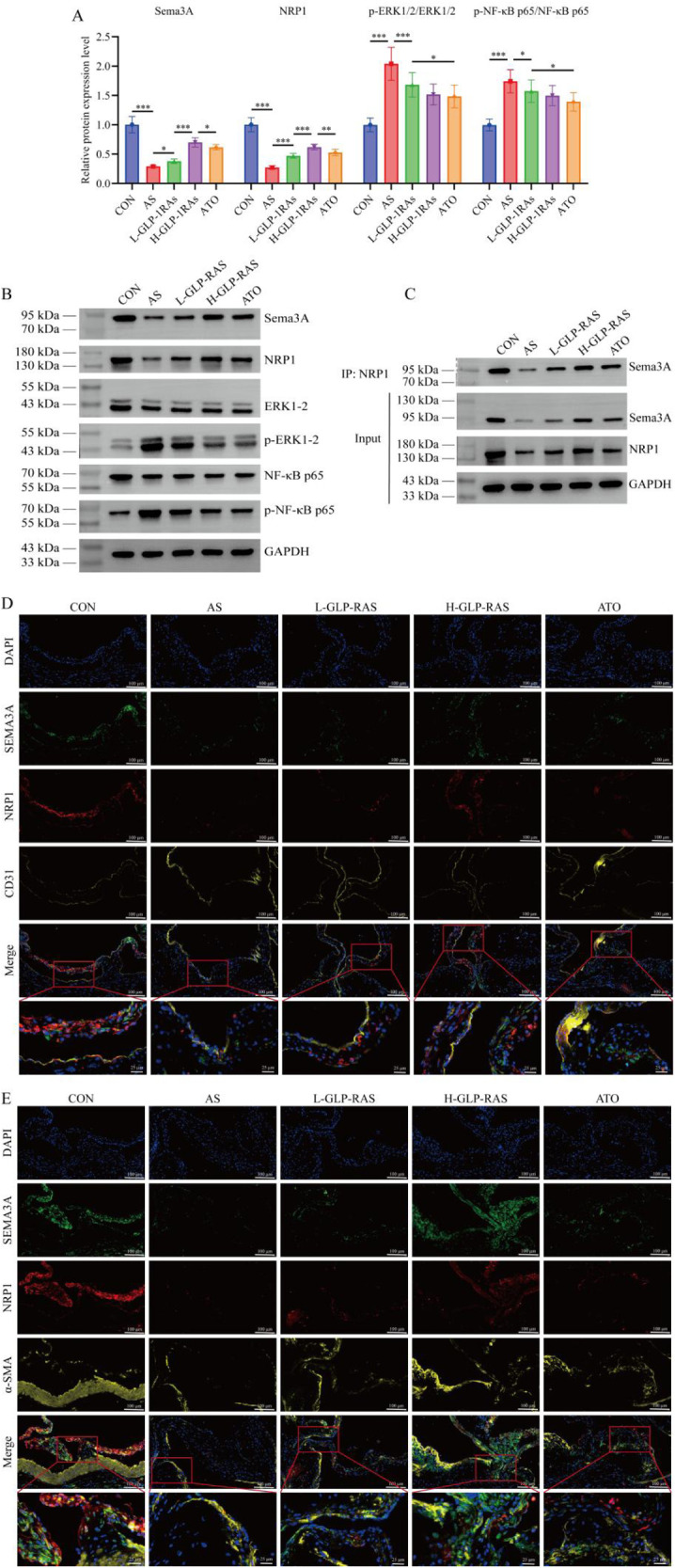
Effects of glucagon-like peptide-1 receptor agonists (GLP-1 RAs) on the semaphorin 3A (Sema3A)/neuropilin-1 (NRP1) pathway and downstream signaling in mouse aorta tissues. **(A,B)** Western blot analysis of Sema3A, NRP1, phosphorylated extracellular signal-regulated kinase 1/2 (p-ERK1/2)/ERK1/2, and p-nuclear factor kappa-B p65 (NF-κB p65)/NF-κB p65 protein expression levels in aortic tissues. **(C)** Co-immunoprecipitation assay to detect Sema3A-NRP1 protein binding in aortic tissues. **(D,E)** Triple immunofluorescence labeling to assess the colocalization of Sema3A and NRP1 with the endothelial cell marker cluster of differentiation 31 (CD31) and the smooth muscle cell marker alpha smooth muscle actin (α-SMA). All data are presented as mean ± SD. *n* = 10. CON, control group; AS, atherosclerotic (AS) model group; L-GLP-1 RAs, low-dose GLP-1 RAs (semaglutide) group; H-GLP-1 RAs, high-dose GLP-1 RAs (semaglutide) group; ATO, atorvastatin group. *P < 0.05, **P < 0.01, ***P < 0.001.

WB analysis (Figures 2A,B) revealed that compared to the CON group, MOD group exhibited significantly reduced Sema3A and NRP1 expression in aortic tissues (*P* < 0.001), alongside significantly increased p-ERK1/2/ERK1/2 and p-NF-κB p65/NF-κB p65 ratios (*P* < 0.001). After treatment with GLP-1 RAs, Sema3A and NRP1 levels were significantly upregulated (*P* < 0.05), while the p-ERK1/2/ERK1/2 ratio was markedly decreased (*P* < 0.05). In the regulation of Sema3A and NRP1, high-dose GLP-1 RAs demonstrated more pronounced effects compared to low-dose GLP-1 RAs and ATO (*P* < 0.001). Co-IP results (Figure 2C) demonstrated that Sema3A-NRP1 binding in aortic tissues of atherosclerotic mice was lower compared to normal mice, whereas GLP-1 RAs treatment increased the binding level. IF colocalization (Figures 2D,E) confirmed that Sema3A and NRP1 mainly localized in endothelial cells.

### 3.3 GLP-1 RAs improve vascular endothelial cell function and suppress inflammation in ox-LDL-induced HUVECs

**Figure 3:**
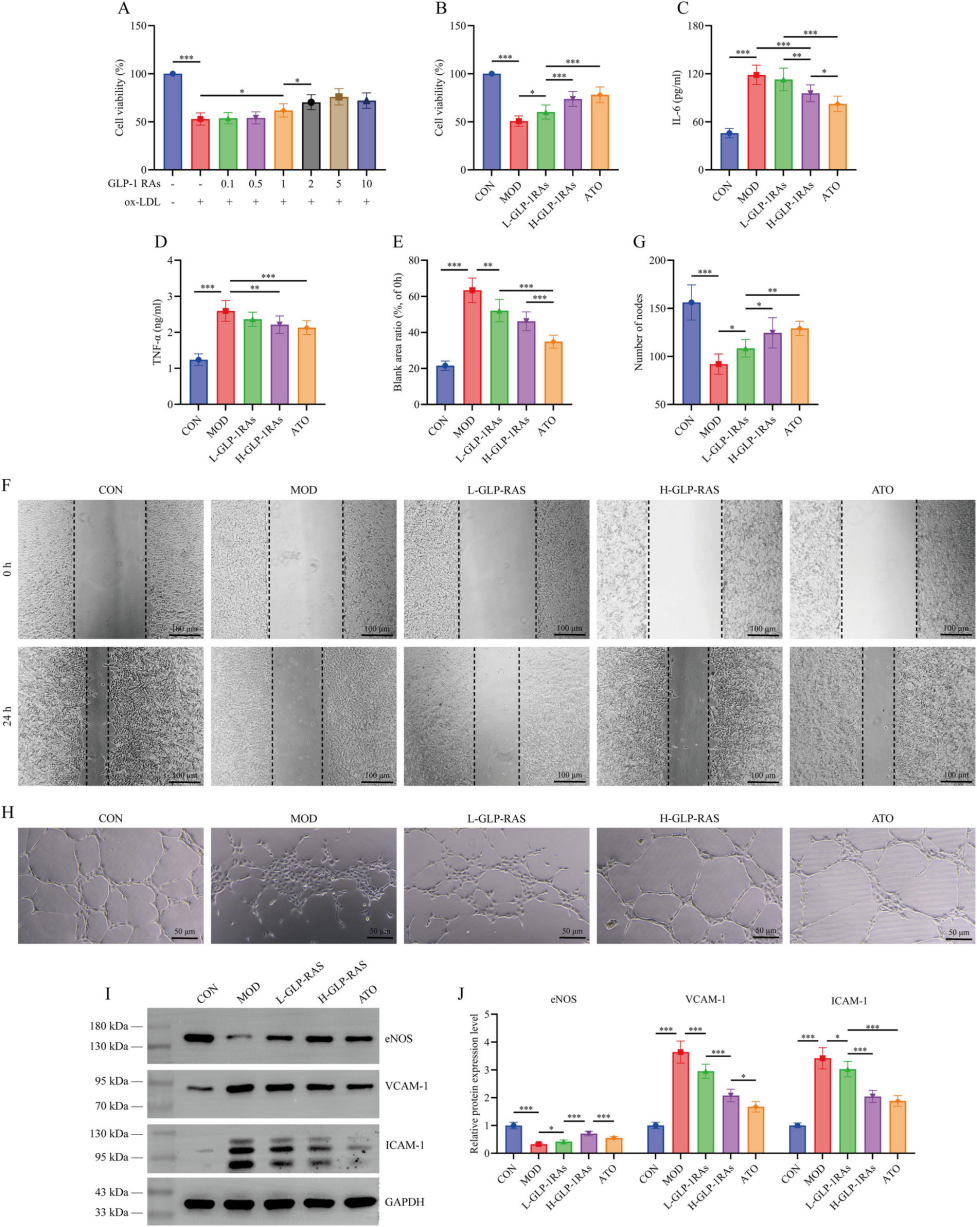
Effects of glucagon-like peptide-1 receptor agonists (GLP-1 RAs) on oxidized low-density lipoprotein (ox-LDL)-induced inflammation and dysfunction in vascular endothelial cells. **(A)** Human umbilical vein endothelial cells (HUVECs) were treated with 0.1, 0.5, 1, 2, 5, or 10 μM GLP-1 RAs (semaglutide) to assess cell viability and determine low and high doses of semaglutide. **(B)** Cell viability assay. **(C)** IL-6 levels in cell culture supernatants. **(D)** TNF-α levels in cell culture supernatants. **(E,F)** Wound healing assay. **(G,H)** Tube formation assay. **(I,J)** Western blot analysis of endothelial nitric oxide synthase (eNOS), vascular cell adhesion molecule-1 (VCAM-1), and intercellular adhesion molecule-1 (ICAM-1) protein expression levels in cells. All data are presented as mean ± SD. *n* = 6. CON, control group; ox-LDL, ox-LDL treated group; L-GLP-1 RAs, low-dose GLP-1 RAs (semaglutide) group; H-GLP-1 RAs, high-dose GLP-1 RAs (semaglutide) group; ATO, atorvastatin group. *P < 0.05, **P < 0.01, ***P < 0.001.

The CCK-8 assay results in Figure 3A showed the treatment of ox-LDL-induced HUVECs with semaglutide at concentrations of 0.1, 0.5, 1, 2, 5, 10 μM. At semaglutide concentrations of 1, 2, 5 and 10 μM, HUVECs cell viability was significantly higher compared to the MOD group (*P* < 0.05). However, when the semaglutide concentration increased to 10 μM, no further dose-dependent enhancement in viability was observed (*P* > 0.05). Based on these results, 1 μM semaglutide was selected as the experimental dose for the L-GLP-1 RAs group and 2 μM for the H-GLP-1 RAs group. Figures 3B–H demonstrated that in the ox-LDL-induced HUVECs injury model, high dose GLP-1 RAs and ATO interventions significantly improved cell viability, migration capacity, and tube node number (*P* < 0.05), while simultaneously decreased IL-6 and TNF-α levels (*P* < 0.01). WB analysis (Figures 3I,J) revealed that ox-LDL induction markedly suppressed eNOS expression (*P* < 0.001) and upregulated VCAM-1 and ICAM-1 expression (*P* < 0.001). The changes in the expression of the above factors were significantly reversed following GLP-1 RAs and ATO treatments (*P* < 0.05).

### 3.4 GLP-1 RAs upregulate Sema3A/NRP1 expression and binding levels in HUVECs and inhibit downstream ERK1/2 signaling activation

**Figure 4:**
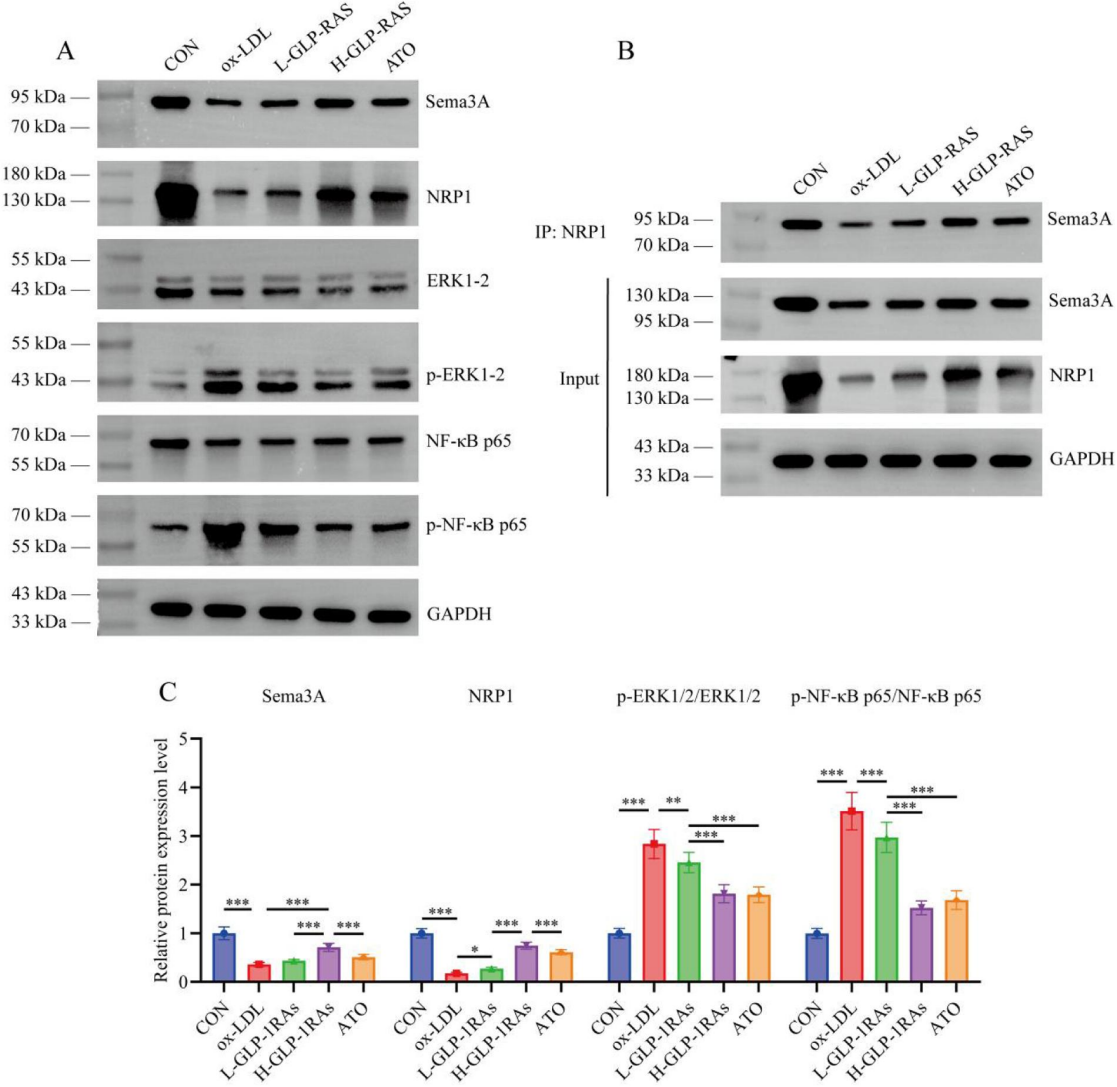
Effects of glucagon-like peptide-1 receptor agonists (GLP-1 RAs) on the semaphorin 3A (Sema3A)/neuropilin-1 (NRP1) pathway and downstream signaling in HUVECs. **(A,B)** Western blot analysis of Sema3A, NRP1, phosphorylated extracellular signal-regulated kinase 1/2 (p-ERK1/2)/ERK1/2, and p-nuclear factor kappa-B p65 (NF-κB p65)/NF-κB p65 protein expression levels in HUVECs. **(C)** Co-immunoprecipitation assay to detect Sema3A-NRP1 protein binding in HUVECs. All data are presented as mean ± SD. *n* = 6. CON, control group; ox-LDL, oxidized low-density lipoprotein (ox-LDL) treated group; L-GLP-1 RAs, low-dose GLP-1 RAs (semaglutide) group; H-GLP-1 RAs, high-dose GLP-1 RAs (semaglutide) group; ATO, atorvastatin group. *P < 0.05, **P < 0.01, ***P < 0.001.

WB and CO-IP results showed that ox-LDL treatment reduced Sema3A and NRP1 expression (*P* < 0.001) and Sema3A-NRP1 binding, while significantly increased p-ERK1/2/ERK1/2 and p-NF-κB p65/NF-κB p65 ratios (*P* < 0.001) in HUVECs (Figures 4A,B). After treatment with GLP-1 RAs, all the above changes were significantly reversed (*P* < 0.05). The effect of H-GLP-1 RAs group exhibited significantly greater improvements than L-GLP-1 RAs group (*P* < 0.001).

### 3.5 Sema3A/NRP1 pathway is an essential factor for GLP-1 RAs-mediated vascular protective effects *in vitro*

**Figure 5:**
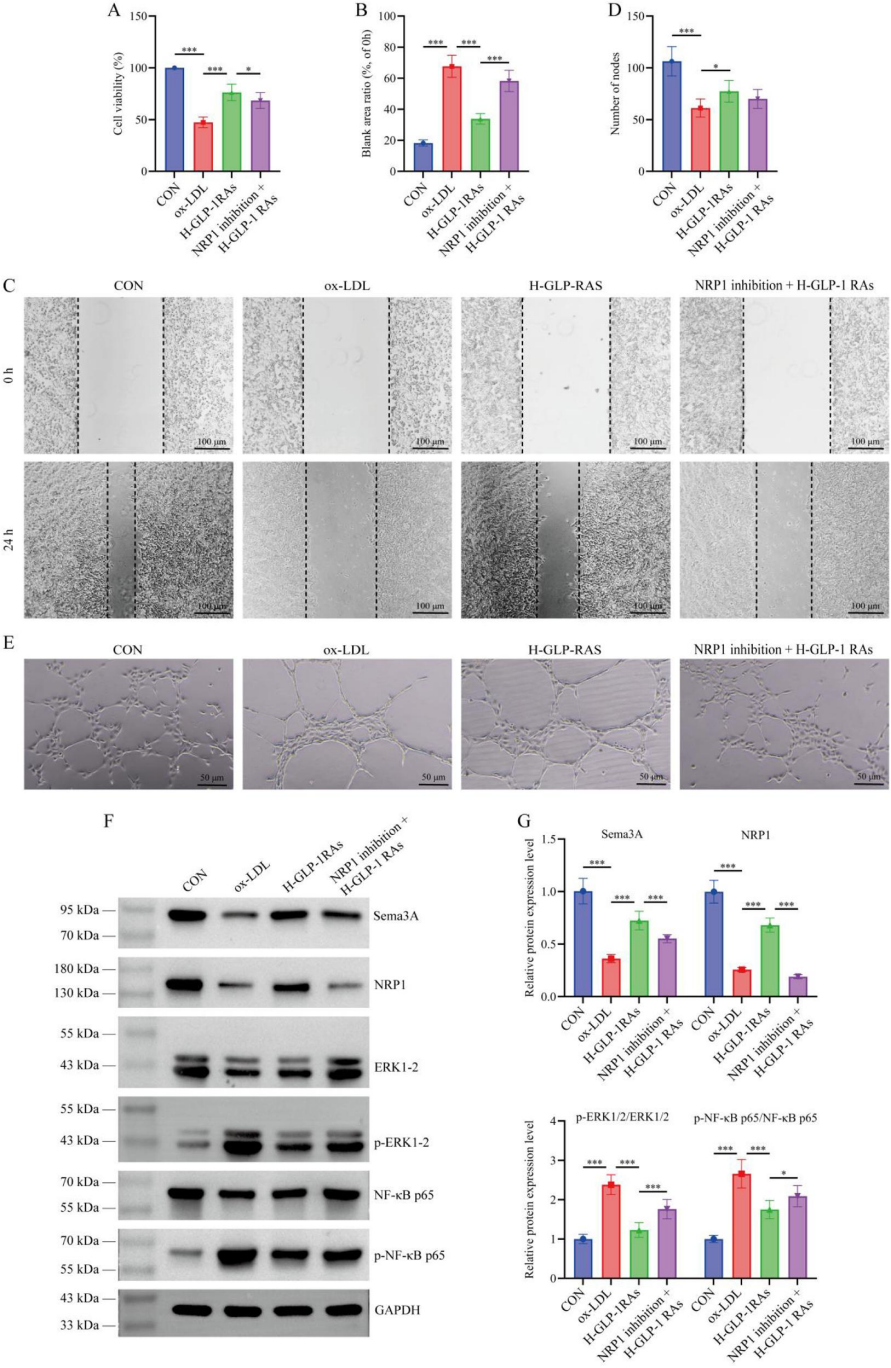
Rescue experiments confirmed the necessity of the emaphorin 3A (Sema3A)/neuropilin-1 (NRP1) pathway in the *in vitro* effects of glucagon-like peptide-1 receptor agonists (GLP-1 RAs). **(A)** Cell viability assay. **(B,C)** Wound healing assay. **(D,E)** Tube formation assay. **(F,G)** Western blot analysis of Sema3A, NRP1, phosphorylated extracellular signal-regulated kinase 1/2 (p-ERK1/2)/ERK1/2, and p-nuclear factor kappa-B p65 (NF-κB p65)/NF-κB p65 protein expression levels in HUVECs. All data are presented as mean ± SD. *n* = 6. CON, control group; ox-LDL, oxidized low-density lipoprotein (ox-LDL) treated group; H-GLP-1 RAs, high-dose GLP-1 RAs (semaglutide) group; NRP1 inhibition + H-GLP-1 RAs, NRP1 inhibitor (EG01377) and high-dose GLP-1 RAs (semaglutide) combined treatment group. *P < 0.05, **P < 0.01, ***P < 0.001.

Figures 5A–E showed that EG01377 significantly reversed the improvements in cell viability, migration capacity and tube formation induced by GLP-1 RAs in ox-LDL-treated HUVECs (*P* < 0.05). Meanwhile, WB results in Figures 5F,G revealed that EG01377 markedly restored the reduced p-ERK1/2/ERK1/2 and p-NF-κB p65/NF-κB p65 ratios caused by GLP-1 RAs treatment (*P* < 0.05).

### 3.6 Vascular endothelial-specific NRP1 knockout confirms the critical role of Sema3A/NRP1 pathway in GLP-1 RAs-mediated alleviation of AS

**Figure 6:**
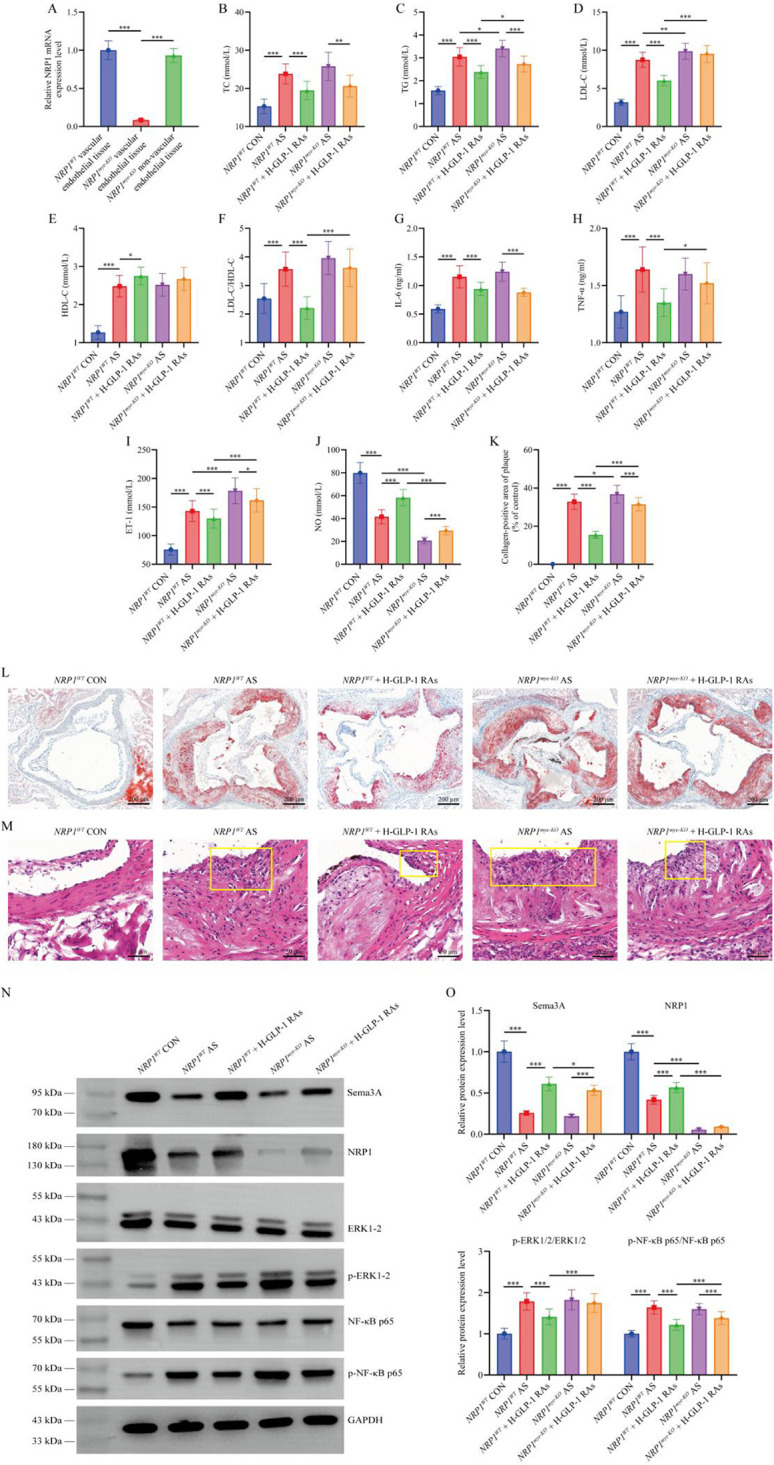
Generation of endothelium-specific neuropilin-1 (NRP1) knockout (*NRP1^EC-KO^*) mice demonstrates the critical role of the emaphorin 3A (Sema3A)/NRP1 signaling pathway in glucagon-like peptide-1 receptor agonists (GLP-1 RAs) alleviating atherosclerosis (aS). **(A)** Quantitative real-time polymerase chain reaction analysis of *NRP1* mRNA expression level in vascular endothelial and non-vascular endothelial tissues. **(B)** Mouse triglycerides (TG) level. **(C)** Mouse total cholesterol (TC) level. **(D)** Mouse low-density lipoprotein cholesterol (LDL-C) level. **(E)** Mouse high-density lipoprotein cholesterol (HDL-C) level. **(F)** Mouse LDL-C/HDL-C ratio. **(G)** Mouse interleukin-6 (IL-6) level. **(H)** Mouse tumor necrosis factor-alpha (TNF-α) level. **(I)** Mouse endothelin-1 (ET-1) level. **(J)** Mouse nitric oxide (NO) level. **(K)** Percentage of collagen-positive area in mouse plaque. Collagen-positive area (%) = (collagen-positive area/plaque area) × 100%. **(L)** Oil red O staining of mouse aortic sinus. **(M)** Hematoxylin and eosin staining of mouse aortic sinus. The area outlined by the yellow box shows inflammatory cell infiltration. **(N,O)** Western blot analysis of Sema3A, NRP1, phosphorylated extracellular signal-regulated kinase 1/2 (p-ERK1/2)/ERK1/2, and p-nuclear factor kappa-B p65 (NF-κB p65)/NF-κB p65 protein expression levels in aortic tissues. All data are presented as mean ± SD. *n* = 10. *NRP1^WT^* CON: *NRP1^WT^* control group; *NRP1^WT^* AS: *NRP1^WT^* AS group; *NRP1^WT^* + H-GLP-1 RAs: *NRP1^WT^* + high-dose GLP-1 RAs (semaglutide) group; *NRP1^EC-KO^* AS: *NRP1^EC-KO^* AS group; *NRP1^EC-KO^* + H-GLP-1 RAs: *NRP1^EC-KO^* + high-dose GLP-1 RAs (semaglutide) group. *P < 0.05, **P < 0.01, ***P < 0.001.

qPCR analysis confirmed the successful model construction, showing virtually no *NRP1* mRNA expression in the vascular endothelial tissues of *NRP1^EC-KO^* mice, while non-endothelial tissues (represented by liver tissue) exhibited comparable *NRP1* mRNA levels to those in *NRP1^WT^* mice (*P* > 0.05, Figure 6A). Figures 6B–J showed that compared with the *NRP1^WT^* model group, the levels of TG, LDL-C and ET-1 in mice of the *NRP1^EC-KO^* model group were increased (*P* < 0.05), and the level of NO was significantly decreased (*P* < 0.001). Oil red O and H&E staining results (Figures 6K–M) revealed that *NRP1^EC-KO^* model mice displayed higher collagen-positive area percentages in plaque (*P* < 0.05) and more severe inflammatory infiltration (outlined in yellow) compared to *NRP1^WT^* model mice. These findings indicate that endothelial NRP1 deficiency may exacerbates AS. Among the 3 groups of *NRP1^WT^* mice, the changing trends of all the above indicators were consistent with observations in Sections 3.1 and 3.2, where high-dose GLP-1 RAs significantly improved atherosclerotic phenotypes (*P* < 0.05). However, in *NRP1^EC-KO^* mice, the therapeutic effects of high-dose GLP-1 RAs were significantly weakened. TG, LDL-C/HDL-C, TNF-α, ET-1 levels, plaque collagen-positive area percentages, p-ERK1/2/ERK1/2, and p-NF-κB p65/NF-κB p65 ratios remained significantly higher in *NRP1^EC-KO^* + H-GLP-1 RAs group compared to *NRP1^WT^* + H-GLP-1 RAs group (*P* < 0.05). Additionally, NO levels and the expression of Sema3A and NRP1 were significantly lower (*P* < 0.05), and aortic inflammatory infiltration showed no notable improvement.

## 4 Discussion

The pathological process of AS is complex, mainly characterized by endothelial dysfunction and persistent inflammatory responses that drive atherosclerotic plaque formation and progression (19). Current targeted interventions for inflammation and endothelial dysfunction remain insufficient, necessitating the exploration of novel regulatory mechanisms and therapeutic targets. As a class of novel antidiabetic drugs, GLP-1 RAs have been validated by multiple trials to exert clear anti-atherosclerotic effects (20). However, the specific molecular mechanisms underlying these effects remain incompletely elucidated, limiting their application and optimization in AS prevention and treatment. This study systematically investigates the molecular mechanisms by which GLP-1 RAs alleviate inflammatory responses and endothelial dysfunction in AS via the Sema3A/NRP1 pathway.

*ApoE^−/−^* mice induced by high-fat diet exhibit significant weight gain, dyslipidemia and inflammatory factor release, which makes them a classical model for clinical AS research (21). The model group mice in this study showed similar changes in related indicators, indicating successful establishment of the AS model. This study also found that GLP-1 RAs could improve pathological phenotypes, restore endothelial function, and suppress inflammatory responses in atherosclerotic mice. These effects include reductions in body weight, lipid levels, inflammatory factors, and ET-1 levels, increased NO levels, reduced plaque collagen-positive area, and decreased inflammatory cell infiltration. These findings are consistent with previous studies. Existing animal experiments have confirmed that GLP-1 RAs can reduce body weight, blood glucose and lipid levels in Sprague-Dawley rats (22, 23), and inhibit serum inflammatory markers in obese mice (24, 25). Additional preclinical studies indicate that GLP-1 RAs exert direct effects on vascular endothelium to alleviate vascular dysfunction (26). A study in rats demonstrated that liraglutide can mediate vasodilation under conditions of reduced ET-1 levels, thereby improving coronary microcirculation (27). The above results of this study further confirm that the anti-atherosclerotic effects of GLP-1 RAs operate through dual mechanisms: lipid metabolism regulation and inflammatory response suppression, potentially independent of their hypoglycemic effects. The regulation of mouse body weight by GLP-1 RAs may also exert indirect cardiovascular protective effects by reducing vascular metabolic burden. This corresponds with the findings of Galasso et al. (28), which indicate that GLP-1 RAs not only have hypoglycemic effects but also can improve fat accumulation and metabolic parameters. Although this study did not examine vasodilation in mice, it did assess endothelial-related factors, specifically ET-1 and NO levels. The significant improvement in both of both factors were consistent with the trends observed in the studies by Liu (29) and Jonik et al. (30). In addition, this study observed that the effects of high-dose GLP-1 RAs were comparable to those of the positive control drug atorvastatin, with only a relatively significant difference exists in the improvement of collagen-positive plaque area. This provides experimental evidence supporting GLP-1 RAs as a potential alternative therapeutic approach for AS.

On this basis, this study explored the regulatory effect of GLP-1 RAs on the Sema3A/NRP1 pathway and found that GLP-1 RAs can upregulate the expression of Sema3A and NRP1 in aortic tissues of atherosclerotic mice, enhance their binding levels, and simultaneously inhibit the activation of downstream ERK1/2 and NF-κB signaling pathways. This finding is consistent with previous research on the vascular protective functions of the Sema3A/NRP1 pathway. Existing studies indicates that the Sema3A/NRP1 pathway is a critical regulatory pathway for vascular homeostasis and can regulate pathological neovascularization (31, 32). Clinical studies indicate that Sema3A protein expression levels in AS patients are significantly lower than in healthy controls (6). In animal models exhibiting atherosclerotic plaques, Sema3A regulates endothelial cell apoptosis by activating the downstream Nrp/plexin signaling pathway, thereby further modulates angiogenesis (33). The ERK1/2-NF-κB signaling axis is a key pathway mediating inflammatory responses and vascular cell injury (34). Wu et al. (9) reported that Sema3A primarily suppresses aortic angiogenesis and inflammation by binding to NRP1 on endothelial cells and inhibiting downstream ERK signaling. Previous studies have identified that GLP-1 RAs can inhibit the ERK and NF-κB pathways, exerting protective effects in type 2 diabetes and obesity (35, 36). However, this study firstly associates the regulation of GLP-1 RAs with the Sema3A/NRP1 pathway, elaborates the multiple mechanisms by which GLP-1 RAs influence vascular homeostasis, subsequently modulate downstream inflammatory pathways, and exert cardiovascular protective effects. This study further confirmed through IF that Sema3A and NRP1 are localized in both smooth muscle and endothelial cells. This result is consistent with the study by Wu et al. (9). Other studies have also shown that NRP1 can be expressed by various cell types, including neurons, endothelial cells, smooth muscle cells, and immune cells (8). However, our results indicate that Sema3A and NRP1 are more predominantly localized in vascular endothelial cells.

To further verify the cellular effects of this regulatory mechanism, experiments were conducted using an ox-LDL-induced HUVECs injury model. The results confirmed that GLP-1 RAs could improve cell viability, migration capacity, and tubule formation ability in damaged HUVECs, while reducing IL-6 and TNF-α levels. These findings align with the trends observed *in vivo* experiments and corroborate previous studies demonstrating that GLP-1 RAs possess cytoprotective (37) and anti-inflammatory (38) properties. Endothelial dysfunction involves decreased NO bioavailability resulting from reduced eNOS expression, as well as enhanced leukocyte recruitment and inflammatory responses driven by elevated VCAM-1 and ICAM-1 levels (39). This study also found that GLP-1 RAs can reverse the decreased expression of eNOS and the increased expression of VCAM-1 and ICAM-1 induced by ox-LDL. Similar observations were reported by Oh et al. (40), who noted that liraglutide alleviated eNOS phosphorylation-related injuries in HUVECs under high-glucose conditions. A study by Punjabi et al. (41) showed that liraglutide reduced vascular endothelial VCAM-1 level in atherosclerotic mice independently of glucose regulation. Another clinical study pointed out that ICAM-1 levels in diabetic patients with AS were higher than those in healthy controls, and semaglutide treatment improved glycemic control, blood pressure, and liver steatosis biomarkers but further elevated ICAM-1 levels (42). Although this outcome is contrary to the findings of this study, both results confirm that GLP-1 RAs therapy modulates molecular changes that are conducive to cardiovascular risk control and vascular endothelial homeostasis.

To determine whether the Sema3A/NRP1 pathway is essential for the protective effects of GLP-1 RAs, this study conducted *in vitro* rescue experiments using the NRP1-specific inhibitor EG01377. Results showed that inhibiting NRP1 function significantly reversed the protective effects of GLP-1 RAs, including improvements in cell viability, migration capacity, tubule formation ability, and suppression of the ERK/NF-κB pathway. This result confirms the indispensability of the Sema3A/NRP1 pathway in the *in vitro* vascular protective effects of GLP-1RAs. To further verify the role of this pathway at the genetic level *in vivo*, endothelial-specific NRP1 knockout (*NRP1^EC-KO^*) mice were generated. Previous study using this model have confirmed that endothelial-specific NRP1 deficiency exacerbates atherosclerotic pathology in hypercholesterolemic mice without affecting survival (43). In this study, *NRP1^EC-KO^* mice exhibited more severe dyslipidemia, endothelial dysfunction, and inflammatory infiltration, which confirms the endogenous protective role of vascular endothelial NRP1. It is well established that endothelial NRP1 is crucial for maintaining the integrity of the vascular endothelial barrier (7). Damage to the vascular endothelial barrier can accelerate the infiltration and deposition of circulating lipids into the vascular intima (44). We hypothesize that the loss of NRP1 in endothelial cells exacerbates endothelial dysfunction, thereby aggravating vascular inflammation and metabolic burden, which in turn disrupts the balance of lipid metabolism. The results of this study also indicate that the effect of GLP-1 RAs in *NRP1^EC-KO^* mice were significantly diminished, showing no notable improvement in pathological damage. Meanwhile, the p-ERK1/2/ERK1/2 ratio were restored to higher levels. These findings were mutually consistent with the *in vitro* rescue experiments, collectively demonstrating from both *in vitro* and *in vivo* perspectives that GLP-1 RAs exert anti-atherosclerotic effects by protecting endothelial cells and suppressing inflammation through the Sema3A/NRP1 pathway.

This study still has certain limitations. For example, only semaglutide was selected as a representative of GLP-1 RAs for experiments, without verifying the regulatory effects of other GLP-1 RAs such as liraglutide and dulaglutide on the Sema3A/NRP1 pathway. Additionally, experiments were conducted in mouse models and HUVECs, lacking validation in diverse cell/animal types or clinical samples from atherosclerotic patients. Future studies could incorporate clinical samples to compare multiple GLP-1 RAs and further explore the regulatory interactions between the Sema3A/NRP1 pathway and other downstream signaling pathways of the GLP-1 receptor. The finding in this study that endothelial NRP1 knockout can influence the lipid profile to a certain extent is also worthy of further validation and investigation of its underlying mechanisms in future studies.

## 5 Conclusion

In conclusion, this study combined *in vivo* and *in vitro* experiments to clarify the molecular mechanism by which GLP-1 RAs alleviate inflammation and endothelial dysfunction in AS through activating the Sema3A/NRP1 pathway and suppressing downstream ERK1/2-NF-κB signaling.

## Data Availability

The original contributions presented in the study are included in the article/Supplementary Material, further inquiries can be directed to the corresponding author.
